# Opioids Switching with Transdermal Systems in Chronic Cancer Pain

**DOI:** 10.1186/1756-9966-28-61

**Published:** 2009-05-07

**Authors:** C Aurilio, MC Pace, V Pota, P Sansone, M Barbarisi, E Grella, MB Passavanti

**Affiliations:** 1 Department of Anesthesiological, Surgical and Emergency Sciences, Second University of Naples, Naples, Italy; 2 Department of Plastic Surgery, Second University of Naples, Naples, Italy

## Abstract

**Background:**

Due to tolerance development and adverse side effects, chronic pain patients frequently need to be switched to alternative opioid therapy

**Objective:**

To assess the efficacy and tolerability of an alternative transdermally applied (TDS) opioid in patients with chronic cancer pain receiving insufficient analgesia using their present treatment.

**Methods:**

A total of 32 patients received alternative opioid therapy, 16 were switched from buprenorphine to fentanyl and 16 were switched from fentanyl to buprenorphine. The dosage used was 50% of that indicated in equipotency conversion tables. Pain relief was assessed at weekly intervals for the next 3 weeks

**Results:**

Pain relief as assessed by VAS, PPI, and PRI significantly improved (p < 0.0001) in all patients at all 3 follow up visits. After 3 weeks of treatment, the reduction in the mean VAS, PPI, and PRI scores in the fentanyl and buprenorphine groups was 68, 77, 74, and 69, 79, and 62%, respectively. Over the same time period the use of oral morphine as rescue medication was reduced from 27.5 ± 20.5 (mean ± SD) to 3.75 ± 8.06, and 33.8 ± 18.9 to 3.75 ± 10.9 mg/day in the fentanyl and buprenorphine groups, respectively. There was no significant difference in either pain relief or rescue medication use between the two patient groups The number of patient with adverse events fell during the study. After the third week of the treatment the number of patients with constipation was reduced from 11 to 5, and 10 to 4 patients in the fentanyl and buprenorphine groups, respectively. There was a similar reduction in the incidence of nausea and vomiting. No sedation was seen in any patient after one week of treatment.

**Conclusion:**

Opioid switching at 50% of the calculated equianalgesic dose produced a significant reduction in pain levels and rescue medication. The incidence of side effects decreased and no new side effects were noted. Further studies are required to provide individualized treatment for patients according to their different types of cancer.

## Introduction

Opioids represent the principal therapy in chronic moderate to severe cancer pain treatment. The development of transdermal polymer matrix systems for opioid administration has resulted in several advantages compared to oral, sublingual or parenteral administration. These systems represent a non-invasive method, effective and well accepted by cancer patients who often have gastrointestinal problems and difficulties with oral medication (e.g. oesophageal, gastric, intestinal or maxillofacial cancer) either due to the cancer itself or due to the side-effects on oral or parenteral concomitant medication [1]. At present, the opioids buprenorphine and fentanyl are available in transdermal formulations.

Recent studies have shown that opioid transdermal delivery systems have numerous advantages since they permit continuous controlled release of the opioid for 72, or even up to 96 hours depending on the product, thus reducing peaks in plasma drug concentrations resulting in consistent and long-term pain relief. In addition, they are associated with a lower rate of adverse events. Overall, they represent a very useful therapy since they offer adequate analgesia with comparably low side-effects and non-invasive administration. However, analgesic tolerance can develop with any long-term opioid treatment, requiring an increase in drug dosage in order to obtain the same analgesic effect. As a consequence this normally results in an increase in side effects [2,3]. In cases where patients are not achieving satisfactory analgesia, or are suffering from intolerable side-effects, the guidelines of the World Health Organization for cancer pain treatment recommend switching to an alternative opioid. For many patients opioid switching or rotation is the only solution for pain relief [4,5]. Prior to the introduction of a new formulation it is necessary to establish an approximate dose ratio to provide an equivalent analgesic effect.

Considering the importance of this strategy, we carried out this study on opioid switching using two polymer matrix systems: transdermal buprenorphine (BTDS) and transdermal fentanyl (FTDS) substituting the opioid previously taken with the other type (e.g. FTDS if they were originally taking BTDS, and vice versa) in patients who were dissatisfied with their previous therapy with respect to inadequate analgesia, side-effects or both. Based on previously published data and considering the mechanisms which form the basis of tolerance phenomena, the aim of this study was to evaluate the switching dose between transdermal opioids, with regard to analgesic efficacy and the reduction of side-effects.

## Patients and methods

### Patients

Eligible patients, of either sex, were suffering from chronic pain and had been treated for the previous three months with either transdermal buprenorphine or transdermal fentanyl. Inclusion criteria required inadequate analgesia (Visual Analogue Scale [VAS] > 50 mm, and the presence of adverse events correlating with opioid analgesic treatment (sedation, dysphoria, nausea/vomiting and constipation).

Exclusion criteria included renal insufficiency (serum creatinine clearance less than 60 ml/min), moderate or severe hepatic disease (Child-Pugh score between 7 and 10 or between 10 and 15, respectively), history of hepatitis B or C, or acute hepatitis A in the last three months, HIV, clinically significant cardiovascular and/or respiratory diseases, pregnancy, lactation, alcohol consumption, psychotropic drug consumption. Also excluded were patients expecting chemotherapy or radiotherapy cycles during the study, those with known allergy to the medicines or matrix patch components, those with extensive skin disease, and patients who were currently participating in, or had within the last 30 days participated in, other clinical trials. Patients with neurological and/or psychological conditions that might hinder completing daily diaries and pain scales were also excluded.

### Study procedure

The study was carried out according to the ethical principles of the current amended version of Declaration of Helsinki, after ethics committee approval. All the patients gave their signed informed consent before participation in the study.

The four-week study was organized with a Screening visit (V0) followed by a Recruitment visit (V1) one week later, when treatment was initiated. Three control visits (V2, V3, and V4) at weekly intervals then followed.

During the screening visit (V0) the age, sex and race of each patient was noted and a detailed history of the cancer disease and of the concomitant pain was taken. Each patient underwent a thorough a physical examination including height, weight and vital signs (blood pressure, respiratory frequency and heart rate). The presence or absence of other concomitant disease and their treatment was registered. Haematochemical analyses were carried out to evaluate hepatic and renal function (Transaminase, Electrolytes, Urea, Creatinine, Cholinesterase, Prothrombin and Partial Thromboplastin time, International Normalised Ratio) (Cr, NA, K, BU, GPT, GOT, γGT, CHE, PT, aPTT, INR). An ECG was performed together with a neurological examination. During the visit the type of transdermal patch and the dose were noted.

At the end of the screening visit the patients were discharged and told to continue the previous therapy. They were asked to return to the department for the recruitment visit one week later. All the patients received a diary in which to rate their pain every morning on awakening on a VAS scale. Patients were permitted to continue with rescue medication (20 mg oral immediate release (IR) morphine) up to a maximum of three daily doses, which was recorded in the diary.

During the recruitment visit each patient underwent a thorough physical examination: general appearance, eyes, lungs, heart, abdomen, musculoskeletal and vital signs were evaluated. The results of haematochemical examinations for renal and hepatic function and the results of the neurological and cardiological examinations were recorded. Adverse Events (AEs) were evaluated. The consumption of rescue medication in mg/day was recorded.

Patients complying with the inclusion criteria were divided into two groups according to the administered therapy up to the recruitment visit. The method used for transdermal patch switching was to replace the first opioid patch with the alternative one, deducting 50% from the dose calculated according to equianalgesic tables.

The FTDS group were patients at the screening visit who had taken buprenorphine TDS 70 μg/h and suffered side-effects and refractory pain and had taken this dose continuously during the pre-recruitment week. At the recruitment visit, the transdermal buprenorphine patch in these patients was replaced by a 25 μg/h transdermal fentanyl patch, positioned at different skin site on the thorax, arm or back.

The BTDS group were patients at the screening visit who had taken fentanyl TTS 75 μg/h and suffered side-effects and refractory pain and had taken this dose continuously during the pre-recruitment week. The transdermal fentanyl patch in these patients was replaced by a 52.5 μg/h transdermal buprenorphine patch, positioned at different skin site on the thorax, arm or back.

Rescue medication with 20 mg of immediate-release oral morphine was prescribed to each patient up to three times a day. At the end of the recruitment visit (V1) all the patients were asked to return after one week for the first control visit (V2), and to continue keeping their daily diaries.

### Assessment of analgesic efficacy

Mean weekly pain on the basis of the VAS scores in diaries (VAS 0 = no pain to VAS 100 = intolerable pain) was recorded throughout the 4 week period. The Present Pain Intensity (PPI, 0 = no pain, 1 = mild, 2 = discomforting, 3 = distressing, 4 = horrible, 5 = excruciating) and Pain Rating Index (PRI) were assessed during each visit from V1 to V4. The PRI was taken from the Short-Form Mc Gill Pain Questionnaire and comprised 15 items investigating both the sensorial (11 items) and the emotional sphere of pain (4 items) with a score from 0 to 3 for each item (0–45). In all cases the necessity of rescue medication was registered as milligrams of oral morphine per day. Another parameter taken into consideration was the patients' satisfaction with the new therapy. It was evaluated by means of the simple question: "Are you satisfied with your analgesic treatment?" The patients could answer only "Yes/No".

The primary efficacy measure was pain reduction as recorded by patients both in a daily dairy using VAS and during the visits by PPI and PRI. The secondary efficacy measure was the reduction of rescue mediation consumption as milligrams of IR oral morphine per day.

### Assessment of adverse events

In all patients, the presence (Yes) or absence (No) of AEs was evaluated and recorded in response to questions posed for nausea and/or vomiting, constipation, and dysphoria. The level of sedation was evaluated by a 4-point scale (0 = no sedation, 1 = slight sedation, 2 = moderate sedation, 3 = severe sedation).

### Statistical analysis

For each of the two treatment groups, a paired Student t test was used to compare the mean values of the primary efficacy parameters (VAS, PPI, and PRI) and rescue medication consumption for the same patients measured at Visits 2, 3, and 4 compared to baseline values (Visit 1). A Student t test for independent variables was used to compare the two independent treatment groups.

## Results

In total, 40 Caucasian patients were screened and 32 were enrolled. All the enrolled patients completed the study. The demographic characteristics of the patients are shown in table 1.

**Table 1:** Demographic Data of Enrolled Patients_[SN6]_

Sex	
Male	17
Female	15
	
Age	
Mean	62
*Range*	*42–78*
	
Cancer Site	
Lung	8
Colon	2
Stomach	5
Bladder	1
Breast	1
Prostate	11
Gall Bladder	2
Brain primitive cancer	2

There were no differences in vital signs and no respiratory depression was noted in either group. No significant differences were showed between Group BTDS and Group FTDS regarding VAS, PPI, PRI values, AEs incidence and rescue medication consumption on enrolment.

### Analgesic efficacy

**Figure 1:**
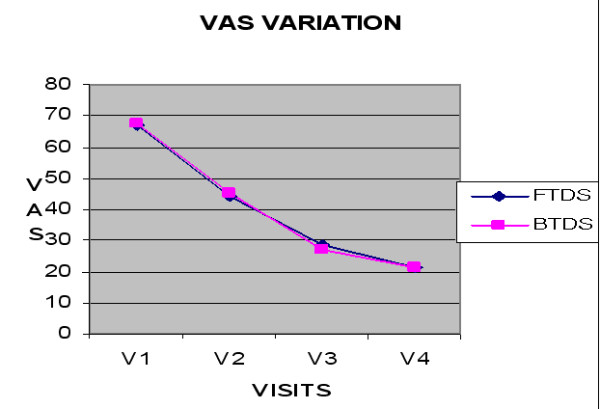
**Mean Weekly VAS**.

In both groups of patients, there was a statistically significant reduction (p < 0.0001) of the weekly VAS after 1, 2 and 3 weeks treatment compared to V1 values. The mean decrease in the FDTS group was 34% (V2), 57% (V3) and 68% (V4), and in the BTDS group was 33% (V2), 60% (V3), 69% (V4) (table 2 and figure 1). The was no statistically significant difference between the two groups at any visit.

**Table 2:** 

	**Mean VAS ± SD**				**Mean PPI ± SD**				**Mean PRI ± SD**			
	V1	V2	V3	V4	V1	V2	V3	V4	V1	V2	V3	V4
**FTDS**	66.9 ± 14.0	44.4 ± 14.1	28.8 ± 13.6	21.2 ± 12.0	3.50 ± 0.89	1.62 ± 0.72	1.0 ± 0.63	0.81 ± 0.66	32.4 ± 2.13	24.2 ± 6.46	14.4 ± 4.01	11.6 ± 1.59
% reduction from V1		34	57	68		54	71	77		35	66	74
p		<0.0001	<0.0001	<0.0001		<0.0001	<0.0001	<0.0001		<0.0001	<0.0001	<0.0001
**BTDS**	67,5 ± 13,4	45.0 ± 11.5	26.9 ± 10.8	21.2 ± 13.6	3.5 ± 0.82	1.44 ± 0.63	0.88 ± 0.81	0.75 ± 0.86	33.1 ± 1.91	22.1 ± 7.18	18.3 ± 4.66	12.5 ± 1.97
% reduction fromV1		33	60	69		59	75	79		43	45	62
P		<0.0001	<0.0001	<0.0001		<0.0001	<0.0001	<0.0001		<0.0001	<0.0001	<0.0001

**Figure 2:**
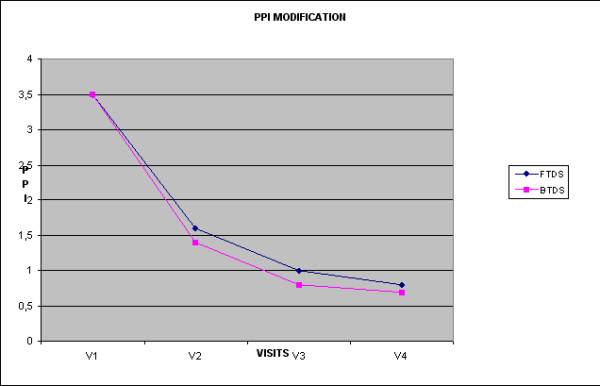
**Mean Weekly PPI**.

In both groups of patients, there was a statistically significant reduction in the PPI score (p < 0.0001) at each visit after commencing treatment. The mean decrease in the FTDS group was 54% (V2), 71% (V3), and 77% (V4), and in the BTDS group 59% (V2), 75% (V3), and 79% (V4) (table 2 and figure 2). There was no statistically significant difference between the two groups at any visit.

**Figure 3:**
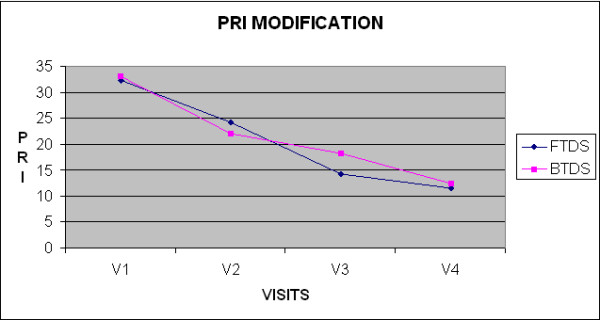
**Mean Weekly PRI**.

A significant reduction was also observed in PRI. (p < 0.0001) as showed in table 2 and figure 3. The mean decrease in the FTDS group was 35% (V2), 66% (V3), and 74% (V4), and in the BTDS group 43% (V2), 45% (V3), and 62% (V4). There was no statistically significant difference between the FTDS and BTDS groups at any visit.

**Figure 4:**
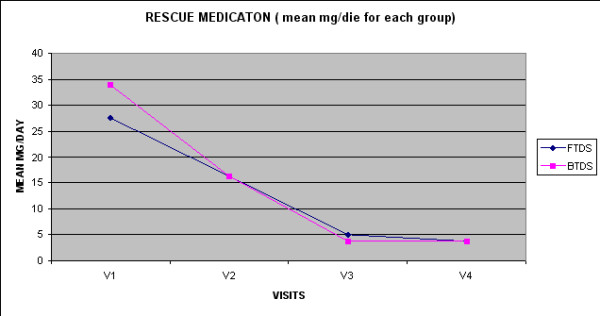
**Mean of daoli milligrams of IR oral Morphine recorded each visit**.

In all patients there was a reduction in rescue medication at Visits 2, 3, and 4 as measured by the daily consumption of IR oral morphine (figure 4). This was statistically significant (p < 0.0001) at V3 and V4 in both treatment groups (Table 3). There was no significant difference between the two patient groups.

**Table 3:** RESCUE MEDICATION (mean mg/day for each group ± SD)

	RESCUE MEDICATION (mean mg/day for each group ± SD)			
	V1	V2	V3	V4
FTDS	27.5 ± 20.5	16.2 ± 18.24	5.0 ± 13.7	3.75 mg ± 8.06
p value compared to V1		0.095	0.002	<0.0001
BTDS	33.8 ± 18.9	16.2 ± 20.9	3.75 ± 10.9	3.75 ± 10.9
p value compared to V1		0.058	<0.0001	<0.0001

Patients of both groups stated that they were satisfied with the therapy, in fact all the patients answered yes to the question: "Are you satisfied with your analgesic treatment?"

### Adverse events

Transdermal opioid switching reduced the incidence of adverse events. Nausea and vomiting persisted in patients suffering from gall bladder cancer and gastric cancer (three patients). The number of patients with constipation was also reduced; BTDS group: V1 11 pts, V2 4 pts, V3 5 pts, V4 5 pts and similarly in the FTDS group: V1 10 pts, V2 6 pts, V3 4 pts, V4 5 pts (table 4 and table 5). Constipation persisted only in patients suffering from colon, brain and lung cancer (9 patients). Moreover, in both groups, dysphoria and sedation disappeared completely after the first week (tables 4 and 5).

**Table 4:** 

	Number of patients with Nausea and/or vomiting				Number of patients with constipation				Number of patients with dysphoria			
	V1	V2	V3	V4	V1	V2	V3	V4	V1	V2	V3	V4
FTDS	9	6	5	3	10	6	4	5	0	0	0	0
BTDS	8	5	4	2	11	4	5	4	2	0	0	0

**Table 5:** SEDATION SCALE

	SEDATION SCALE															
	Number of patients without Sedation				Number of patients with slight sedation				Number of patients with moderate sedation				Number of patients with severe sedation			
	V1	V2	V3	V4	V1	V2	V3	V4	V1	V2	V3	V4	V1	V2	V3	V4
FTDS	10	16	16	16	2	0	0	0	4	0	0	0	0	0	0	0
BTDS	12	16	16	16	3	0	0	0	1	0	0	0	0	0	0	0

## Discussion

Opioid switching is a fundamentally useful strategy in long-term treatment of cancer pain, where tolerance phenomena and the large number of side-effects can limit the use of these medicines and further diminish the patients' quality of life [6,8]. In these cases, switching from one opioid to another is a useful means to establish a more favourable balance between analgesia and toxicity and is regulated in conversion tables in order to ensure fewer side-effects and an improvement in pain symptoms. [7,9,10,12].

The development of tolerance suggests the necessity to increase the drug dose in order to obtain the same analgesic effect [13,14]. Tolerance development may also be associated with pharmacodynamic, pharmacokinetic and psychological processes resulting in an increase in side effects connected not only with the drug, but also with its metabolites. It may be supposed that by changing the opioid and using lower doses than indicated in conversion tables it is possible, in most cases, to reduce toxicity and improve pain symptoms [6,15,16].

According to available data, many factors may influence opioid treatment such as individual variability, genetic factors, relation among active metabolites, intrinsic activity, number and types of receptors, as well as issues of efficacy, toxicity and tolerance. Considering the above cited variables and the importance of an individualized treatment, we decided to switch buprenorphine TDS with fentanyl TDS, and vice versa, in order to utilize the particular characteristics of these two opioids, without changing route of administration [17].

Our data indicated that by switching buprenorphine TDS to fentanyl TDS and vice versa, with a 50% reduction of the new opioid dose over that given in the conversion tables we obtained a significant reduction of both pain and rescue medication. Moreover, side effects decreased and no new side effects became apparent.

Our results are a starting point for further studies and reiterate the importance of providing individualized treatment and taking the site of the cancer into account (the three patients who still had nausea and vomiting had gastric and gall bladder cancer). This applies not only to the therapeutic formulation but also to the side effect analysis, so that we can gain a better understanding of how much the adverse events are connected with the choice of opioid and how much they are related, or supported by, the underlying pathology of the disease.

In our study we decided to change the drug and not the route of administration, because patients prefer a transdermal route as it does not interfere with their daily activities, it is easy to use, and is non invasive. Transdermal route patients only have to remember their opioid medication every 72 hours. Reduced constipation, nausea and vomiting result in a better quality of life. These factors account for better patient compliance and lead to the feeling of greater independency from treatment. All patients stated that they were satisfied with the therapy and this result is particularly important because, as the international literature underlines, psychological factors interfere with patients' quality of life and disease prognosis [13,18-20].

In contrast with our results, other studies discuss the necessity of using equianalgesic doses in opioid switching to obtain good pain control [16]. These differences suggest that the drug, its formulation, individual response and the route of administration are all variables of fundamental importance in the therapeutic result, and that the response to opioids does not depend on the pathophysiology of the pain alone, but rather a complex phenomenon linked to individual factors.

## Conclusion

In conclusion, we think that further studies should be performed in order to find safe and effective opioid switching methods necessary to give greater insight into the difficult balance between analgesia and toxicity. It is also important to consider individual variables, such as psychological distress in cancer patients, as these are important as prognostic factors since they affect therapeutic results.

## Competing interests

The authors declare that they have no competing interests.

## Authors' contributions

A.C. as principal investigator of this study, had full access to all of the data in the study and takes responsibility for the integrity of the data and the accuracy of the data analyses. Study concept and design: A.C., P.M.C. Acquisition of data: S.P., P.V., B.M., G.E. Analysis and interpretation of data: S.P., P.V. Drafting of the manuscript: S.P., P.V., B.M., G.E. Critical revision of the manuscript for important intellectual content: A.C., P.M.C., P.M.B. Study supervision: A.C., P.M.C., P.M.B. All authors read and approved the final manuscripts
